# Supplementary material for: Assessing universality of DNA barcoding in geographically isolated selected desert medicinal species of Fabaceae and Poaceae
Source: PeerJ. 2018 Mar 13;6:e4499. doi: 10.7717/peerj.4499 (PMC5855882; doi:10.7717/peerj.4499)
Supplement: Supplemental Information 1 [file peerj-06-4499-s001.docx]

**Table S1: Supplemental information of specimens, BOLD IDs and accession numbers**

| Species of family Fabaceae | BOLD process ID | GenBank Accession Number | | | Voucher | Collection site and Geo. coordinates | Elevation (m) |
| --- | --- | --- | --- | --- | --- | --- | --- |
|  |  | ITS2 | *mat*K | *rbc*La |  |  |  |
| *Acacia jacquemontii* | DBMPP090-14  DBMPP091-14  DBMPP092-14 | MG270127  MG270128  MG270129 | MF694816 | MF694937 | 822-30-2014  (a,b,c) | Chananpir  28°97'N 71°85'E | 109 |
| *Acacia modesta* | DBMPP089-14  DBMPP102-14  DBMPP103-14 | MG270130 | MF694817 | MF694938  MF694648  MF694649 | 822-31-2014  (a,b,c) | Derawar fort  28.655, 71.812 | 109.12 |
| *Acacia nilotica* | DBMPP099-14  DBMPP100-14  DBMPP101-14 | MG270131  MG270132 | MF694818 | MF694939  MF694650  MF694651 | 822-32-2014  (a,b,c) | Dingarh  28.963, 71.852 | 109.12 |
| *Crotalaria burhia* | DBMPP109-14  DBMPP110-14  DBMPP111-14 | MG270136  MG270137  MG270138 | ------ | MF694969  MF694695  MF694696 | 822-35-2014  (a,b,c) | Dingarh  28.963, 71.852 | 109.12 |
| *Indigofera hochstetteri* | DBMPP116-14  DBMPP117-14  DBMPP118-14 | MG270142  MG270143  MG270144 | MF694859 | MF694988  MF694731  MF694732 | 822-38-2014  (a,b,c) | Dingarh 28.886, 71.669 | 109.12 |
| *Indigofera heterantha* | DBMPP180-14  DBMPP298-16  DBMPP299-16 | MG270140  MG270141 | MF694858 | MF694987  MF694730 | 822-28-2014  (a,b,c) | Dingarh 28.886, 71.669 | 109.12 |
| *Prosopis cineraria* | DBMPP096-14  DBMPP097-14  DBMPP098-14 | ------- | ------- | MF694762  MF694763 | 822-33-2014  (a,b,c) | Dingarh 28.886, 71.669 | 109.12 |
| Species of family Poaceae | | | | | | | |
| *Cenchrus ciliaris* | DBMPP106-14  DBMPP107-14  DBMPP108-14 | MG270133  MG270134  MG270135 | MF694838 | MF694962  MF694686  MF694683 | 822-36-2014  (a,b,c) | Kanchi mor 29.183, 71.674 | 109.12 |
| *Cymbopogon jawarancusa* | DBMPP104-14  DBMPP105-14  DBMPP119-14 | MG270139  MG256234 | MF694845 | MF694970  MF694697  MF694698 | 822-37-2014  (a,b,c) | Dingarh 29.046, 71.821 | 109.12 |
| *Lasiurus scindicus* | DBMPP113-14  DBMPP114-14  DBMPP115-14 | MG270145  MG270146  MG270147 | MF694862 | MF694991  MF694737  MF694738 | 822-40-2014  (a,b,c) | Chananpir 28.58, 71.42 | 109.12 |
