# Supplementary material for: Assessing universality of DNA barcoding in geographically isolated selected desert medicinal species of Fabaceae and Poaceae
Source: PeerJ. 2018 Mar 13;6:e4499. doi: 10.7717/peerj.4499 (PMC5855882; doi:10.7717/peerj.4499)
Supplement: Supplemental Information 2 [file peerj-06-4499-s002.docx]

**Table S2: Primers**

| **Name** | **Sequence 5′-3′** | **Source** |
| --- | --- | --- |
| ***mat*K**  KIM_3FKIM-r  KIM_1RKIM-f | CGTACAGTACTTTTGTGTTTACGAG  ACCCAGTCCATCTGGAAATCTTGGTTC | Ki-Joong Kim, Pers. comm.  Ki-Joong Kim, Pers. comm. |
| ***rbc*La**  rbcLa-F  rbcLa-R | ATGTCACCACAAACAGAGACTAAAGC  GTAAAATCAAGTCCACCRCG | (Levin, 2003)  (Kress and Erickson, 2007) |
| **ITS2**  ITS2-S2F  ITS4 | ATGCGATACTTGGTGTGAAT  GTAAAATCAAGTCCACCRCG | (Chen et al., 2010)  (White et al., 1990) |

**Table S3: Thermocycler programming for PCR of candidate barcodes**

| **Cycle step and number of cycles** | | ***mat*K** | | ***rbc*La** | | **ITS2** | |
| --- | --- | --- | --- | --- | --- | --- | --- |
|  |  | Block temp. | Hold time (mm:ss) | Block temp. | Hold time (mm:ss) | Block temp. | Hold time (mm:ss) |
| Init. denat. | 1 | 98 ˚C | 00:45 | 94 ˚C | 04:00 | 94 ˚C | 05:00 |
| Denaturation  Annealing  Extension | 35 | 98 ˚C  52 ˚C  72 ˚C | 00:10  00:30  00:40 | 94 ˚C  55 ˚C  72 ˚C | 00:30  00:30  01:00 | 94 ˚C  56 ˚C  72 ˚C | 00:30  00:30  00:45 |
| Final Ext. | 1 | 72 ˚C | 10:00 | 72 ˚C | 10:00 | 72 ˚C | 10:00 |
