# Supplementary material for: Assessing universality of DNA barcoding in geographically isolated selected desert medicinal species of Fabaceae and Poaceae
Source: PeerJ. 2018 Mar 13;6:e4499. doi: 10.7717/peerj.4499 (PMC5855882; doi:10.7717/peerj.4499)
Supplement: Supplemental Information 3 [file peerj-06-4499-s003.docx]

| **Family** | **Barcode region (sample size)** | **Best Match (%)** | | **Best Close Match (%)** | |
| --- | --- | --- | --- | --- | --- |
|  |  | Correct | Ambiguous | Correct | Ambiguous |
| Fabaceae | **ITS2 (18)** | 17 (100) | 0.00 (0) | 16 (94.11) | 0.00 (0) |
|  | ***mat*K (15)** | 15 (100) | 0.00 (0) | 13 (86.66) | 0.00 (0) |
|  | ***rbc*La (21)** | 15 (71.42) | 6 (28.57) | 14 (66.66) | 6 (28.57) |
|  | **ITS2+*mat*K (15)** | 15 (100) | 0.00 (0) | 12 (80) | 0.00 (0) |
|  | **ITS2+*rbc*La (18)** | 18 (100) | 0.00 (0) | 15 (83.33) | 0.00 (0) |
|  | ***mat*K+*rbc*La (15)** | 15 (100) | 0.00 (0) | 11 (73.33) | 0.00 (0) |
|  | **ITS2+*mat*K+*rbc*La (15)** | 15 (100) | 0.00 (0) | 11 (73.33) | 0.00 (0) |
| Poaceae | **ITS2 (9)** | 9 (100) | 0.00 (0) | 9 (100) | 0.00 (0) |
|  | ***mat*K (9)** | 9 (100) | 0.00 (0) | 8 (88.88) | 0.00 (0) |
|  | ***rbc*La (9)** | 9 (100) | 0.00 (0) | 8 (88.88) | 0.00 (0) |
|  | **ITS2+*mat*K (9)** | 9 (100) | 0.00 (0) | 8 (88.88) | 0.00 (0) |
|  | **ITS2+*rbc*La (9)** | 9 (100) | 0.00 (0) | 8 (88.88) | 0.00 (0) |
|  | ***mat*K+*rbc*La (9)** | 9 (100) | 0.00 (0) | 7 (77.77) | 0.00 (0) |
|  | **ITS2+*mat*K+*rbc*La (9)** | 9 (100) | 0.00 (0) | 7 (77.77) | 0.00 (0) |
| Fabaceae + Poaceae | **ITS2 (27)** | 26 (100) | 0.00 (0) | 25 (96.15) | 0.00 (0) |
|  | ***mat*K (24)** | 23 (95.83) | 0.00 (0) | 21 (87.5) | 0.00 (0) |
|  | ***rbc*La (30)** | 24 (80) | 6 (20) | 22 (73.33) | 6 (20) |
|  | **ITS2+*mat*K (24)** | 24 (100) | 0.00 (0) | 20 (83.33) | 0.00 (0) |
|  | **ITS2+*rbc*La (27)** | 27(100) | 0.00 (0) | 23 (85.18) | 0.00 (0) |
|  | ***mat*K+*rbc*La (24)** | 24 (100) | 0.00 (0) | 18 (75.0) | 0.00 (0) |
|  | **ITS2+*mat*K+*rbc*La (24)** | 24 (100) | 0.00 (0) | 18 (75.0) | 0.00 (0) |

**Table S4: Species level discrimination ability of candidate barcodes by BM and BCM analysis**
