# Supplementary material for: Assessing universality of DNA barcoding in geographically isolated selected desert medicinal species of Fabaceae and Poaceae
Source: PeerJ. 2018 Mar 13;6:e4499. doi: 10.7717/peerj.4499 (PMC5855882; doi:10.7717/peerj.4499)
Supplement: Supplemental Information 11 [file peerj-06-4499-s011.doc]

**Figure S1. Evolutionary relationships of taxa with ITS2**

The evolutionary history was inferred using the Neighbor-Joining method . The optimal tree with the sum of branch length = 2.79376101 is shown. The percentage of replicate trees in which the associated taxa clustered together in the bootstrap test (500 replicates) are shown next to the branches. The tree is drawn to scale, with branch lengths in the same units as those of the evolutionary distances used to infer the phylogenetic tree. The evolutionary distances were computed using the Kimura 2-parameter method and are in the units of the number of base substitutions per site. The rate variation among sites was modeled with a gamma distribution (shape parameter = 5). The analysis involved 27 nucleotide sequences. All ambiguous positions were removed for each sequence pair. There were a total of 365 positions in the final dataset. Evolutionary analyses were conducted in MEGA6.

**Figure S2. Molecular Phylogenetic analysis by Maximum Likelihood method with ITS2**

The evolutionary history was inferred by using the Maximum Likelihood method based on the Tamura 3-parameter model. The tree with the highest log likelihood (-2661.1029) is shown. The percentage of trees in which the associated taxa clustered together is shown next to the branches. Initial tree(s) for the heuristic search were obtained automatically by applying Neighbor-Join and BioNJ algorithms to a matrix of pairwise distances estimated using the Maximum Composite Likelihood (MCL) approach, and then selecting the topology with superior log likelihood value. The rate variation model allowed for some sites to be evolutionarily invariable ([+*I*], 12.8371% sites). The tree is drawn to scale, with branch lengths measured in the number of substitutions per site. The analysis involved 27 nucleotide sequences. There were a total of 365 positions in the final dataset. Evolutionary analyses were conducted in MEGA6.

**Figure S3. Evolutionary relationships of taxa with *mat*K**

The evolutionary history was inferred using the Neighbor-Joining method. The optimal tree with the sum of branch length = 0.46015160 is shown. The percentage of replicate trees in which the associated taxa clustered together in the bootstrap test (500 replicates) are shown next to the branches. The tree is drawn to scale, with branch lengths in the same units as those of the evolutionary distances used to infer the phylogenetic tree. The evolutionary distances were computed using the Tamura 3-parameter method and are in the units of the number of base substitutions per site. The rate variation among sites was modeled with a gamma distribution (shape parameter = 5). The analysis involved 24 nucleotide sequences. All positions containing gaps and missing data were eliminated. There were a total of 443 positions in the final dataset. Evolutionary analyses were conducted in MEGA6.

**Figure S4. Molecular Phylogenetic analysis by Maximum Likelihood method with *mat*K**

The evolutionary history was inferred by using the Maximum Likelihood method based on the Tamura 3-parameter model. The tree with the highest log likelihood (-1284.7714) is shown. The percentage of trees in which the associated taxa clustered together is shown next to the branches. Initial tree(s) for the heuristic search were obtained automatically by applying Neighbor-Join and BioNJ algorithms to a matrix of pairwise distances estimated using the Maximum Composite Likelihood (MCL) approach, and then selecting the topology with superior log likelihood value. A discrete Gamma distribution was used to model evolutionary rate differences among sites (5 categories (+*G*, parameter = 0.8074)). The tree is drawn to scale, with branch lengths measured in the number of substitutions per site. The analysis involved 24 nucleotide sequences. All positions containing gaps and missing data were eliminated. There were a total of 443 positions in the final dataset. Evolutionary analyses were conducted in MEGA6.

**Figure S5. Evolutionary relationships of taxa with *rbc*La**

The evolutionary history was inferred using the Neighbor-Joining method. The optimal tree with the sum of branch length = 0.22715459 is shown. The percentage of replicate trees in which the associated taxa clustered together in the bootstrap test (500 replicates) are shown next to the branches. The tree is drawn to scale, with branch lengths in the same units as those of the evolutionary distances used to infer the phylogenetic tree. The evolutionary distances were computed using the Kimura 2-parameter method and are in the units of the number of base substitutions per site. The rate variation among sites was modeled with a gamma distribution (shape parameter = 5). The analysis involved 30 nucleotide sequences. All positions containing gaps and missing data were eliminated. There were a total of 415 positions in the final dataset. Evolutionary analyses were conducted in MEGA6.

**Figure S6. Molecular Phylogenetic analysis by Maximum Likelihood method with *rbc*La**

The evolutionary history was inferred by using the Maximum Likelihood method based on the Kimura 2-parameter model. The tree with the highest log likelihood (-1057.9168) is shown. The percentage of trees in which the associated taxa clustered together is shown next to the branches. Initial tree(s) for the heuristic search were obtained automatically by applying Neighbor-Join and BioNJ algorithms to a matrix of pairwise distances estimated using the Maximum Composite Likelihood (MCL) approach, and then selecting the topology with superior log likelihood value. A discrete Gamma distribution was used to model evolutionary rate differences among sites (5 categories (+*G*, parameter = 0.2172)). The tree is drawn to scale, with branch lengths measured in the number of substitutions per site. The analysis involved 30 nucleotide sequences. All positions containing gaps and missing data were eliminated. There were a total of 415 positions in the final dataset. Evolutionary analyses were conducted in MEGA6.

**Figure S7. Evolutionary relationships of taxa with ITS2+*mat*K**

The evolutionary history was inferred using the Neighbor-Joining method. The optimal tree with the sum of branch length = 0.69514893 is shown. The percentage of replicate trees in which the associated taxa clustered together in the bootstrap test (500 replicates) are shown next to the branches. The tree is drawn to scale, with branch lengths in the same units as those of the evolutionary distances used to infer the phylogenetic tree. The evolutionary distances were computed using the Tamura 3-parameter method and are in the units of the number of base substitutions per site. The rate variation among sites was modeled with a gamma distribution (shape parameter = 5). The analysis involved 24 nucleotide sequences. All positions containing gaps and missing data were eliminated. There were a total of 544 positions in the final dataset. Evolutionary analyses were conducted in MEGA6.

**Figure S8. Molecular Phylogenetic analysis by**

**Maximum Likelihood method with ITS2+*mat*K**

The evolutionary history was inferred by using the Maximum Likelihood method based on the Tamura 3-parameter model. The tree with the highest log likelihood (-2202.4196) is shown. The percentage of trees in which the associated taxa clustered together is shown next to the branches. Initial tree(s) for the heuristic search were obtained automatically by applying Neighbor-Join and BioNJ algorithms to a matrix of pairwise distances estimated using the Maximum Composite Likelihood (MCL) approach, and then selecting the topology with superior log likelihood value. A discrete Gamma distribution was used to model evolutionary rate differences among sites (5 categories (+*G*, parameter = 0.4648)). The tree is drawn to scale, with branch lengths measured in the number of substitutions per site. The analysis involved 24 nucleotide sequences. All positions containing gaps and missing data were eliminated. There were a total of 544 positions in the final dataset. Evolutionary analyses were conducted in MEGA6.

**Figure S9. Evolutionary relationships of taxa with ITS2+*rbc*La**

The evolutionary history was inferred using the Neighbor-Joining method. The optimal tree with the sum of branch length = 0.49913626 is shown. The percentage of replicate trees in which the associated taxa clustered together in the bootstrap test (500 replicates) are shown next to the branches. The tree is drawn to scale, with branch lengths in the same units as those of the evolutionary distances used to infer the phylogenetic tree. The evolutionary distances were computed using the Kimura 2-parameter method and are in the units of the number of base substitutions per site. The rate variation among sites was modeled with a gamma distribution (shape parameter = 5). The analysis involved 27 nucleotide sequences. All positions containing gaps and missing data were eliminated. There were a total of 513 positions in the final dataset. Evolutionary analyses were conducted in MEGA6.

**Figure S10. Molecular Phylogenetic analysis by Maximum Likelihood method with ITS2+*rbc*La**

The evolutionary history was inferred by using the Maximum Likelihood method based on the Kimura 2-parameter model. The tree with the highest log likelihood (-1959.6764) is shown. The percentage of trees in which the associated taxa clustered together is shown next to the branches. Initial tree(s) for the heuristic search were obtained automatically by applying Neighbor-Join and BioNJ algorithms to a matrix of pairwise distances estimated using the Maximum Composite Likelihood (MCL) approach, and then selecting the topology with superior log likelihood value. A discrete Gamma distribution was used to model evolutionary rate differences among sites (5 categories (+*G*, parameter = 0.2306)). The tree is drawn to scale, with branch lengths measured in the number of substitutions per site. The analysis involved 27 nucleotide sequences. All positions containing gaps and missing data were eliminated. There were a total of 513 positions in the final dataset. Evolutionary analyses were conducted in MEGA6.

**Figure S11. Evolutionary relationships of taxa with *mat*K+*rbc*La**

The evolutionary history was inferred using the Neighbor-Joining method. The optimal tree with the sum of branch length = 0.31996472 is shown. The percentage of replicate trees in which the associated taxa clustered together in the bootstrap test (500 replicates) are shown next to the branches. The tree is drawn to scale, with branch lengths in the same units as those of the evolutionary distances used to infer the phylogenetic tree. The evolutionary distances were computed using the Tamura 3-parameter method and are in the units of the number of base substitutions per site. The rate variation among sites was modeled with a gamma distribution (shape parameter = 5). The analysis involved 24 nucleotide sequences. All positions containing gaps and missing data were eliminated. There were a total of 858 positions in the final dataset. Evolutionary analyses were conducted in MEGA6.

**Figure S12. Molecular Phylogenetic analysis by Maximum Likelihood method with *mat*K+*rbc*La**

The evolutionary history was inferred by using the Maximum Likelihood method based on the Tamura 3-parameter model. The tree with the highest log likelihood (-2301.0834) is shown. The percentage of trees in which the associated taxa clustered together is shown next to the branches. Initial tree(s) for the heuristic search were obtained automatically by applying Neighbor-Join and BioNJ algorithms to a matrix of pairwise distances estimated using the Maximum Composite Likelihood (MCL) approach, and then selecting the topology with superior log likelihood value. A discrete Gamma distribution was used to model evolutionary rate differences among sites (5 categories (+*G*, parameter = 0.6123)). The tree is drawn to scale, with branch lengths measured in the number of substitutions per site. The analysis involved 24 nucleotide sequences. All positions containing gaps and missing data were eliminated. There were a total of 858 positions in the final dataset. Evolutionary analyses were conducted in MEGA6.

**Figure S13. Evolutionary relationships of taxa with ITS2+*mat*K+*rbc*La**

The evolutionary history was inferred using the Neighbor-Joining method. The optimal tree with the sum of branch length = 0.44710514 is shown. The percentage of replicate trees in which the associated taxa clustered together in the bootstrap test (500 replicates) are shown next to the branches. The tree is drawn to scale, with branch lengths in the same units as those of the evolutionary distances used to infer the phylogenetic tree. The evolutionary distances were computed using the Tamura 3-parameter method and are in the units of the number of base substitutions per site. The rate variation among sites was modeled with a gamma distribution (shape parameter = 5). The analysis involved 24 nucleotide sequences. All positions containing gaps and missing data were eliminated. There were a total of 957 positions in the final dataset. Evolutionary analyses were conducted in MEGA6.

**Figure S14. Molecular Phylogenetic analysis by Maximum Likelihood method with ITS2+*mat*K+*rbc*La**

The evolutionary history was inferred by using the Maximum Likelihood method based on the Tamura 3-parameter model. The tree with the highest log likelihood (-3191.4490) is shown. The percentage of trees in which the associated taxa clustered together is shown next to the branches. Initial tree(s) for the heuristic search were obtained automatically by applying Neighbor-Join and BioNJ algorithms to a matrix of pairwise distances estimated using the Maximum Composite Likelihood (MCL) approach, and then selecting the topology with superior log likelihood value. A discrete Gamma distribution was used to model evolutionary rate differences among sites (5 categories (+*G*, parameter = 0.3194)). The tree is drawn to scale, with branch lengths measured in the number of substitutions per site. The analysis involved 24 nucleotide sequences. All positions containing gaps and missing data were eliminated. There were a total of 957 positions in the final dataset. Evolutionary analyses were conducted in MEGA6.
